# Association of social isolation and loneliness with the incidence and life expectancy of chronic kidney disease: a population-based cohort study

**DOI:** 10.1097/JS9.0000000000003488

**Published:** 2025-09-23

**Authors:** Yanan Guo, Xuechao Li, Yanli Liu, Yanling Lv, Mengyun Luo, Feng Geng, Liangkai Chen, Yujun Wang

**Affiliations:** a Department of Intensive Care Unit, The Central Hospital of Wuhan, Tongji Medical College, Huazhong University of Science and Technology, Wuhan, China; b Department of Urology, The Central Hospital of Wuhan, Tongji Medical College, Huazhong University of Science and Technology, Wuhan, China; c Department of Nutrition and Food Hygiene, Hubei Key Laboratory of Food Nutrition and Safety, School of Public Health, Tongji Medical College, Huazhong University of Science and Technology, Wuhan, China; d Ministry of Education Key Lab of Environment and Health, School of Public Health, Tongji Medical College, Huazhong University of Science and Technology, Wuhan, China; e Prevention Research Collaboration, Sydney School of Public Health, the University of Sydney, New South Wales, Australia Charles Perkins Centre, the University of Sydney, New South Wales, Australia; f Key Laboratory of Vascular Aging, Ministry of Education, Tongji Hospital, Tongji Medical College, Huazhong University of Science and Technology, Wuhan, China

**Keywords:** chronic kidney disease, cohort study, life expectancy, loneliness, social isolation

## Abstract

**Background:**

Mental health has gained unprecedented attention in recent years, with social isolation and loneliness increasingly recognized as risk factors for cardiometabolic diseases and premature mortality. However, the relationship between these psychosocial factors and the incidence and mortality of chronic kidney disease (CKD) remains inadequately explored.

**Methods:**

We utilized data from the UK Biobank cohort, categorizing participants based on their questionnaire responses into groups reflecting the degree of social isolation (least isolated, moderately isolated, most isolated) and presence or absence of loneliness. Cox proportional hazards models were utilized to evaluate the relationships between social isolation, loneliness, and the incidence and mortality rates of CKD. Additionally, life tables were applied to estimate life expectancy.

**Results:**

We included 406 332 individuals without CKD and 60 331 CKD patients. During a median follow-up of 13.5 years, we documented 24 984 incident cases of CKD. Social isolation (HR 1.21, 95% CI: 1.17–1.26) and loneliness (HR 1.26, 95% CI: 1.21–1.33) were both significantly associated with increased CKD risk. Significant interactions were observed between social isolation and genetic risk (*P* for interaction = 0.008), but no such interaction was found for loneliness (*P* for interaction = 0.383). Among CKD patients, those in the most isolated and loneliness groups demonstrated a 33% and 10% higher risk of mortality, respectively. Moreover, life expectancy was notably reduced in the most isolated group (by 2.04 years in females and 2.98 years in males) and in the loneliness group (by 0.58 years in females and 1.03 years in males).

**Conclusions:**

Social isolation and loneliness were independently associated with an elevated risk of both the incidence and mortality of CKD. These findings highlight the need to integrate strategies targeting social isolation and loneliness into CKD prevention and management.

**HIGHLIGHTS**

- Explored the association between social isolation, loneliness, and the risk of chronic kidney disease (CKD) in the general population.
- Provided insights into whether the genetic risk of CKD modified such an association.
- Assess the association of social isolation and loneliness with mortality and life expectancy among CKD patients.

## Introduction

Chronic kidney disease (CKD) contributes substantially to physical disability, psychosocial distress, reduced life expectancy, and imposes considerable financial burdens on healthcare systems^[1–3]^. The global median prevalence of chronic kidney disease is 9.5% (IQR 5.9–11.7)[4], with projections indicating it will rank as the fifth leading cause of mortality worldwide[5]. The etiology of CKD is complex, involving both genetic predispositions and environmental influences^[6-8]^. Among the diverse environmental determinants, psychological factors have garnered growing academic interest due to their potential influence on the onset and progression of CKD. Individuals diagnosed with severe mental illnesses, such as schizophrenia and bipolar disorder, exhibit an elevated risk of developing CKD. Furthermore, pronounced depressive symptoms are significantly correlated with an accelerated decline in renal function^[9,10]^. These psychological factors are closely linked with social isolation. Individuals under chronic stress may withdraw socially, while prolonged isolation can further worsen mental health, forming a bidirectional loop[11]. Thus, social isolation and loneliness reflect both social and psychological vulnerability, potentially contributing to CKD development via neuroendocrine and inflammatory mechanisms.

Social isolation, characterized by limited social interactions, communication, and participation in activities, results in minimal interpersonal contact. Loneliness, conversely, represents a distressing emotional state arising from a perceived gap between actual and desired social connections^[12,13]^. Approximately 25%of community-dwelling older adults experience social isolation, while 35% of individuals aged 45 years and above report loneliness^[13,14]^. Both social isolation and loneliness are recognized as significant medical and public health concerns, with established links to increased risks of adverse cardiometabolic outcomes and premature mortality^[15–17]^. Recent evidence has suggested that social isolation accelerates the decline of estimated glomerular filtration rate (eGFR) and increases the risk of CKD onset among middle-aged and older adults with normal kidney function in China[18]. Additionally, loneliness may present an even greater risk factor for CKD, especially among individuals with diabetes[19]. The chronic nature of CKD and its treatment further exacerbate the disruption of social roles. Medications and their side effects, along with symptoms such as fatigue and pain, as well as renal replacement therapies like peritoneal dialysis, hemodialysis, and kidney transplantation, significantly limit patient participation in daily activities, thereby increasing their vulnerability to loneliness and social isolation^[20–22]^.

While some studies have proposed the potential impact of social isolation and loneliness on CKD patients^[22,23]^, limited research has focused on their long-term association with mortality risk and life expectancy within this population. Moreover, although genetic and environmental factors are established contributors to CKD incidence and progression[8], the role of genetic predisposition in moderating the association between psychosocial factors and CKD risk remains underexplored. To address these knowledge gaps, this study aimed to examine the association between social isolation, loneliness, and the incidence of CKD in the general population, and investigate the genetic interaction in such association. We further sought to assess the association of social isolation and loneliness with mortality and life expectancy among CKD patients.

## Methods

The statistical analysis plan was preregistered on the Open Science Framework (Identifier: DOI 10.17605/OSF.IO/8XSEK), and the work has been reported in line with the STROCSS criteria[24].

### Study population

The UK Biobank is a large, prospective cohort study designed to investigate the impact of various exposures on health outcomes[25]. The cohort includes over 500 000 participants aged 40–69 years, recruited between 2006 and 2010 from 22 assessment centers across England, Scotland, and Wales[26]. Participants with missing data on social isolation or loneliness (*n* = 35 708) were excluded from the study, resulting in a final sample of 466 663 individuals with valid responses. These participants were stratified into two cohorts according to their baseline CKD status, i.e., 60 331 individuals with CKD and 406 332 without CKD at baseline. Genetic risk analyses were conducted within the non-CKD cohort, further excluding participants without genetic data or those with discrepancies between genetic sex and self-reported gender (*n* = 10 891). Furthermore, individuals not self-identified as White British (*n* = 21,840) were excluded to ensure compatibility with the genetic instrument used[27]. The final genetic analysis comprised 378 115 participants. A flowchart outlining the inclusion and exclusion criteria is presented in Supplemental Digital Content Figure 1, available at: http://links.lww.com/JS9/F168. Ethical approval for the UK Biobank study was granted by the North West Multi-Center Research Ethics Committee, and all participants provided written informed consent.

### Outcomes ascertainment

The primary outcomes were CKD incidence in participants without baseline CKD and mortality in CKD patients. Baseline and incident CKD was identified through hospital inpatient records, primary care data, self-reported fields, and baseline examination, including eGFR< 60 mL/min/1.73 m^2^, urinary albumin-to-creatinine ratio (UACR) ≥ 30 mg/g. eGFR was calculated by CKD–EPI using serum creatinine (eGFRcr)[28], cystatin C (eGFRcys) or cystatin C-creatinine (eGFRcr-cys) equations as previously reported[29]. The minimum value obtained from these three methods was selected as the criterion for assessing CKD. Diagnoses were coded in accordance with the International Classification of Diseases, Ninth and Tenth Revisions (ICD-9 and ICD-10) and the Fourth Edition of Procedure Codes. Mortality data were obtained from national death registries, including Hospital Episode Statistics for England, Scottish Morbidity Records, and the Patient Episode Database for Wales. Detailed outcome definitions are provided in Supplemental Digital Content Figure 1, available at: http://links.lww.com/JS9/F168.

### Assessment of social isolation and loneliness

The social isolation index was derived using three questions based on the validated Berkman–Syme social network index[30]: (1) “How often do you visit friends or family or have them visit you?” (1 point for less than one friend or family visit per month); (2) “Which of the following (sports club or gym, pub or social club, religious group, adult education class, other group activity) do you engage in once a week or more often?” (1 point for not participating in any social activities at least weekly); and (3) living alone: “Including yourself, how many people are living together in your household?” (1 point for living alone). Scores ranged from 0 to 3 and were categorized as least isolated (0), moderately isolated (1), and most isolated (≥ 2). Loneliness was assessed using two questions adapted from the revised University of California, Los Angeles loneliness scale[31]: (1) “Do you often feel lonely?” (No = 0, Yes = 1) and (2) “How often are you able to confide in someone close to you?” (almost daily to once every few months = 0, never or almost never = 1). The loneliness score ranged from 0 to 2 points and was dichotomized into no-loneliness (0–1) and loneliness (2) groups.

### Polygenic risk score for CKD

Genotyping and quality control methods in the UK Biobank study have been described previously[32]. We selected 263 independent single-nucleotide polymorphisms (SNPs) genome-wide significantly associated with decreased eGFR levels to calculate CKD-PRS[33], as detailed in Supplemental Digital Content Table 2, available at: http://links.lww.com/JS9/F168. The PRS formula was:

$$CKD-PRS\;=\;\left(\sum_{n=1}^{N}\beta_{n}\cdot SNP_{n}\right)\times \frac{N}{sum\;of\;\beta -coefficients}$$

where *β*_n_ represents the effect size and SNP_n_ is the risk allele count for each variant. The raw PRS was not standardized; it was categorized into tertiles for subsequent analyses. A higher score indicated a greater genetic predisposition to CKD risk and we categorized participants into tertiles of genetic risk as low, intermediate, and high. The weighted PRS inherently approximates a normalized distribution due to cumulative SNP effects, achieving comparable properties to explicit standardization. The PRS was constructed using SNPs significantly associated with eGFR, a continuous measure of kidney function, rather than SNPs associated with clinical CKD diagnosis

To reduce confounding from population stratification, we limited genetic analyses to White British participants, identified by self-reported ethnicity and genetic principal components, and adjusted for the top 10 principal components and genotyping array. This combined approach aligns with standard UK Biobank practices and helps minimize bias from ancestral heterogeneity.

### Assessment of covariates

Baseline covariates included self-reported age, sex (female/male), ethnicity (White/non-White), assessment center (22 categories), Townsend deprivation index (quintiles), physical activity level (low/moderate/high/unknown, quantified via the validated International Physical Activity Questionnaire), smoking status (never/former/current/unknown), alcohol consumption frequency (never or special occasions only/one to three times a month/ once or twice a week/three or four times a week/daily or almost daily/unknown), healthy diet score (a higher score indicates healthier dietary habits, as we previously constructed, Supplemental Digital Content Table 3, available at: http://links.lww.com/JS9/F168)^[34,35]^, body mass index (BMI, categorized as <25.0, 25.0–30.0, and >30.0 kg/m^2^), and medical history (depression, hypertension, diabetes, and cardiovascular disease (CVD). For genetic analyses, CKD-PRS (in tertiles), the first ten principal genetic components, and genotype measurement batches were included as covariates.

### Statistical analysis

We employed Cox proportional hazard regression models to estimate hazard ratios (HR) and 95% confidence interval (CI) for the association of loneliness and social isolation with outcomes (incident CKD and mortality). The proportional hazards assumption was verified using Schoenfeld residuals. Time to event was defined as the duration from baseline to the first-time CKD diagnosis, death, or censorship (31 October 2022 for England, 31 July 2021 for Scotland, and 28 February 2018 for Wales). Three models were employed: Model 1 included age, sex, ethnicity, education, Townsend deprivation index, and assessment centers; Model 2 additionally adjusted for physical activity, smoking status, alcohol consumption, healthy diet score, and BMI; Model 3 further adjusted for depression, dyslipidemia, hypertension, diabetes, CVD, CKD-PRS, genotyping array, and the first 10 principal components of ancestry. Joint analyses examined combinations of social isolation and loneliness categories. Additionally, we conducted genetic interaction analyses to examine the modifying effect of CKD-PRS tertiles on the association between loneliness, social isolation, and the incidence of CKD. We clarify that participants with missing data on key exposures or outcomes were excluded. For covariates, we applied dummy variable adjustment and additionally performed sensitivity analyses, excluding all participants with missing covariate data. To reduce the potential for reverse causality, we excluded participants who developed CKD within the first 2 years of follow-up, as undiagnosed or preclinical CKD at baseline may have influenced reported levels of social isolation or loneliness. For patients with CKD, we applied Cox proportional hazards regression models to estimate the association of social isolation and loneliness with all-cause mortality. To quantify the impact of these exposures on lifespan, we incorporated life expectancy as an additional outcome measure, with the calculation methodology detailed in Supplementary Methods.

We performed subgroup analyses to investigate potential effect modification by sex, age, BMI, Townsend deprivation index, smoking status, alcohol consumption, and physical activity levels. To ensure the robustness of our findings, we performed several sensitivity analyses. These included excluding incident CKD or deaths occurring within the first 2 years of follow-up and utilizing the Fine–Gray subdistribution hazard model to account for competing risks of mortality.

Statistical analyses were performed using SAS software (version 9.4, SAS Institute) and R software (version 4.4.1). A two-sided *P*-value <0.05 was considered statistically significant.

## Results

### Baseline characteristics

Among 406 332 participants without CKD (mean age 56.1 ± 8.1 years, 46.3% male), 163 730 (40.3%) were moderately isolated, 56 959 (14.0%) were most isolated, and 24 999 (6.2%) reported experiencing loneliness (Table 1). Among 60 331 CKD patients (mean age 59.4 ± 7.6 years, 39.4% male), 24 848 (41.9%) were moderately isolated, 9965 (16.5%) were most isolated, and 4423 (7.3%) reported loneliness (Supplemental Digital Content Table 4, available at: http://links.lww.com/JS9/F168). Participants with higher levels of social isolation or loneliness were more likely to be male, have lower socioeconomic status, engage in health-risk behaviors such as smoking and physical inactivity, and have a higher prevalence of depression, hypertension, diabetes, dyslipidemia, and CVD (Table 1, Supplemental Digital Content Tables 5 and 6, available at: http://links.lww.com/JS9/F168).

**Table 1:** The main characteristics of participants by social isolation and loneliness categories

		Social isolation			Loneliness	
Variables	Overall	Least isolated	Moderately isolated	Most isolated	No loneliness	Loneliness
Number of participants, *n* (%)	406 332	185 643 (46.7)	163 730 (40.3)	56 959 (14.0)	381 333 (93.8)	24 999 (6.2)
Age, mean (SD), years	56.1 (8.1)	56.4 (8.1)	55.9 (8.1)	55.5 (7.9)	56.1 (8.1)	55.4 (7.9)
Male, *n* (%)	188 111 (46.3)	84 217 (45.4)	74 876 (45.7)	29 018 (51.0)	176 072 (46.2)	12 039 (48.2)
White ethnicity, *n* (%)	386 878 (95.2)	178 346 (96.1)	155 493 (95.0)	53 039 (93.1)	363 690 (95.4)	23 188 (92.8)
College or university degree, *n* (%)	137 011 (33.7)	62 536 (33.7)	54 719 (33.4)	19 756 (34.7)	130 578 (34.2)	6433 (25.7)
BMI, mean (SD), kg/m^2^	27.3 (4.6)	27.2 (4.4)	27.3 (4.7)	27.5 (5.0)	27.2 (4.6)	28.1 (5.2)
Townsend deprivation index, median (IQR)	–2.2(–3.7, 0.3)	–2.6 (–3.9, 0.5)	–2.0 (–3.6, 0.6)	–1.2 (–3.2, 1.9)	–2.3 (–3.7, 0.2)	–1.3 (–3.2, 1.9)
Current smoker, *n* (%)	40 919 (10.1)	14 523 (7.8)	17 609 (10.8)	8787 (15.3)	36 738 (9.6)	4181 (16.7)
Daily or almost daily alcohol consumption, *n* (%)	85 458 (21.0)	41 011 (22.1)	33 468 (20.4)	10 979 (19.3)	80 833 (21.2)	4625 (18.5)
Healthy diet score, median (IQR)	3.0 (2.0, 4.0)	3.0 (2.0, 4.0)	3.0 (2.0, 4.0)	3.0 (2.0, 4.0)	3.0 (2.0, 4.0)	3.0 (2.0, 4.0)
Physical activity(IPAQ), *n* (%)						
Low	61 663 (15.2)	22 713 (12.2)	26 927 (16.5)	12 023 (21.1)	56 975 (14.9)	4688 (18.8)
Median	137 131 (33.7)	62 307 (33.6)	55 314 (33.8)	19 510 (34.3)	129 503 (34.0)	7628 (30.5)
High	137 214 (33.8)	69 945 (37.8)	52 289 (32.0)	14 980 (26.3)	129 680 (34.0)	7534 (30.1)
Unknown	70 324 (17.3)	30 678 (16.5)	29 200 (17.8)	10 446 (18.3)	65 175 (17.1)	5 149 (20.6)
History of depression, *n* (%)	45 969 (11.3)	18 409 (9.9)	19 453 (11.9)	8107 (14.2)	40 702 (10.7)	5267 (21.1)
History of CVD, *n* (%)	25 079 (6.2)	11 089 (6.0)	10 015 (6.1)	3975 (7.0)	22 812 (6.0)	2267 (9.1)
History of hypertension, *n* (%)	217 215 (53.5)	98 939 (53.3)	87 274 (53.3)	31 002 (54.4)	203 571 (53.4)	13 644 (54.6)
History of diabetes, *n* (%)	20 434 (5.0)	8122 (4.4)	8555 (5.2)	3757 (6.6)	18 557 (4.9)	1877 (7.5)
History of dyslipidemia, *n* (%)	209 256 (51.5)	93 687 (50.5)	84 745 (51.8)	30 824 (54.1)	194 971 (51.1)	14 285 (57.1)

BMI: Body Mass Index; CKD: chronic kidney disease; CVD: cardiovascular disease; IPAQ: International Physical Activity Questionnaire; IQR: interquartile range; SD: standard deviation.

### Association between social isolation, loneliness, and incident CKD

During a median follow-up of 13.5 years (interquartile range: 12.7–14.1), we documented 24 984 new-onset CKD cases. In Model 3, participants categorized as moderately or most isolated had a significantly higher risk of CKD compared with the least isolated group (moderately isolated: HR 1.04, 95% CI: 1.02–1.07, *P* = 0.002; most isolated: HR 1.09, 95% CI: 1.04–1.13, *P* < 0.0001; Table 2). Similarly, participants who reported loneliness had a 12% higher risk of CKD (HR 1.12, 95% CI: 1.08–1.19, *P* < 0.0001) compared with those not reporting loneliness.

**Table 2:** Associations of social isolation and loneliness with the incidence of CKD (*n* = 406 332)

	N	Cases/Person-years	Model 1, HR (95% CI)	Model 2, HR (95% CI)	Model 3, HR (95% CI)
Social isolation					
Least isolated	185 643	10 811/2 406 540	1.00 (Reference)	1.00 (Reference)	1.00 (Reference)
Moderately isolated	163 730	10 265/2 109 946	1.09 (1.06, 1.12)	1.05 (1.02, 1.08)	1.04 (1.02, 1.07)
Most isolated	56 959	3908/725 391	1.21 (1.17, 1.26)	1.10 (1.05, 1.14)	1.09 (1.04, 1.13)
Loneliness					
No	381 333	23 086/4 923 275	1.00 (Reference)	1.00 (Reference)	1.00 (Reference)
Yes	24 999	1898/318 602	1.26 (1.21, 1.33)	1.18 (1.12, 1.23)	1.12 (1.08, 1.19)

Model 1, adjusted for age, sex, ethnicity, education, Townsend deprivation index, and assessment centers.

Model 2, adjusted for covariates in model 1 plus physical activity, smoking status, alcohol consumption, healthy diet score, and body mass index.

Model 3, plus model 2 and history of depression, history of CVD, history of hypertension, history of diabetes, history of dyslipidemia, CKD polygenic risk score, genotyping array, and the first 10 principal components of ancestry. CKD: chronic kidney disease; CVD: cardiovascular disease.

**Figure 1.**
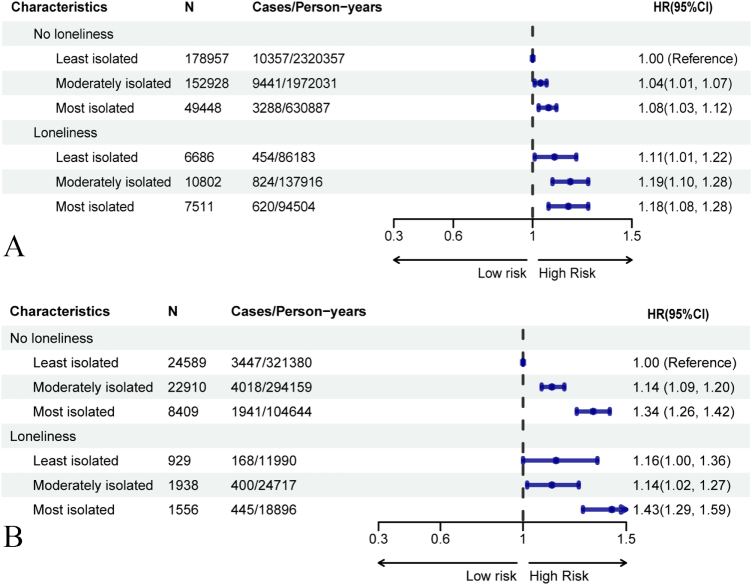
Joint association of social isolation and loneliness with incident and mortality of CKD. (A) CKD incidence; (B) CKD mortality. Reference group: least isolated & not lonely. Models adjusted for demographics, lifestyle factors, comorbidities, and genetic covariates.

The joint association of social isolation and loneliness on incident CKD is shown in Fig. 1(a). Participants reporting both the highest levels of social isolation and loneliness demonstrated the greatest risk of CKD (HR 1.18, 95% CI: 1.08–1.28, *P* = 0.0002) compared to those with the least isolation and no loneliness. However, no significant interaction was observed between social isolation and loneliness in relation to CKD incidence (*P* for interaction = 0.802, Supplemental Digital Content Tables 7 and 8, available at: http://links.lww.com/JS9/F168). Depression partially mediated the associations between social isolation, loneliness, and incident CKD, with the highest proportion mediated observed in the loneliness pathway (9.2%), although the overall mediation effects were modest (Supplemental Digital Content Tables 19, available at: http://links.lww.com/JS9/F168).

**Figure 2.**
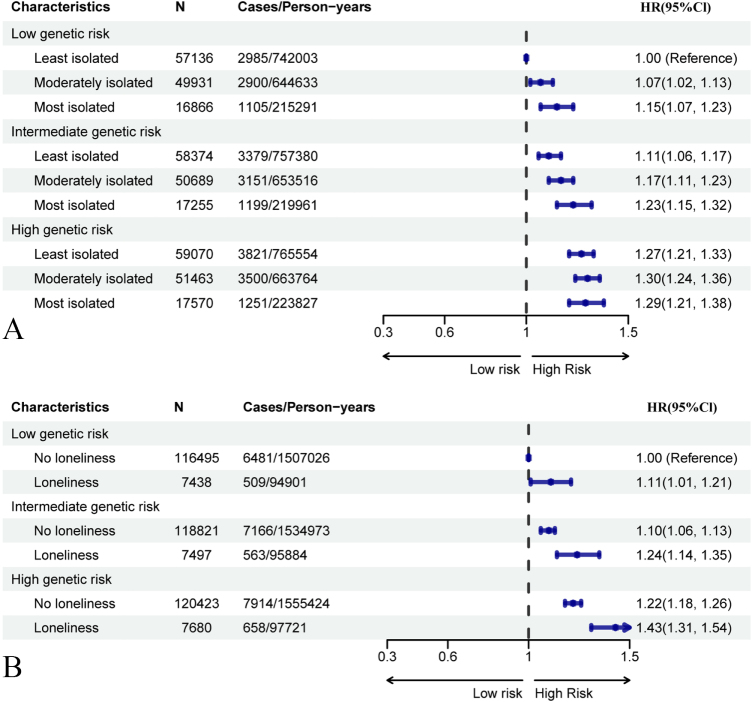
Joint association of social isolation, loneliness and genetic risk with incident CKD. (A) Social isolation × genetic risk; (B) Loneliness × genetic risk. Reference group: low genetic risk & least isolated/not lonely. Models adjusted as in Figure 1.

We observed that social isolation and loneliness were associated with an increased risk of CKD across all CKD-PRS tertiles (Supplemental Digital Content Table 9, available at: http://links.lww.com/JS9/F168). Notably, a significant genetic interaction was observed only for social isolation (*P* for interaction = 0.008), but not for loneliness (*P* for interaction = 0.383). In joint analyses, participants with both the highest levels of social isolation and elevated genetic risk exhibited the greatest risk of CKD (HR 1.29, 95% CI: 1.21–1.38, *P* < 0.0001) compared to all other groups (Fig. 2). Similarly, individuals experiencing loneliness in combination with high genetic risk showed an even more pronounced risk of CKD (HR 1.43, 95% CI: 1.31–1.54, *P*< 0.0001) compared to those without loneliness and with low genetic risk.

### Association of social isolation and loneliness with mortality and life expectancy in CKD patients

**Figure 3.**
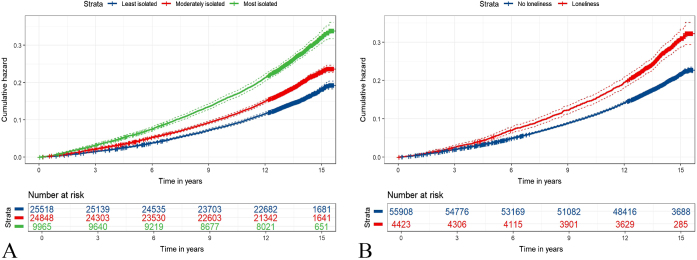
The cumulative risks of mortality of CKD according to the loneliness and social isolation status. (A) Cumulative risk of CKD mortality by social isolation status. (B) Cumulative risk of CKD mortality by loneliness status. CKD, chronic kidney disease.

Kaplan–Meier survival curves demonstrated significant differences in mortality risk among CKD patients based on social isolation and loneliness levels (Fig. 3). The most isolated patients had a 33% higher risk of mortality compared to the least isolated group (HR 1.33, 95% CI: 1.26–1.40, *P* < 0.0001), while those with moderate isolation had a 12% increased mortality (HR 1.12, 95% CI: 1.07–1.17, *P* < 0.0001). CKD patients reporting loneliness had a 10% higher risk of mortality compared to those without loneliness (HR 1.10, 95% CI: 1.03–1.17, *P* = 0.007; Table 3). The joint association of social isolation and loneliness with mortality is presented in Fig. 1(b). Depression showed limited mediation in the associations between social isolation and CKD mortality (1.2–1.9%), but played a more notable mediating role in the link between loneliness and mortality, with 12.75% of the effect mediated (Supplemental Digital Content Tables 20, available at: http://links.lww.com/JS9/F168).

**Table 3:** Associations of social isolation and loneliness with CKD mortality (*n* = 60 331)

	N	Cases/Person-years	Model 1, HR (95% CI)	Model 2, HR (95% CI)	Model 3, HR (95% CI)
Social isolation					
Least isolated	25 518	3615/333 370	1.00 (Reference)	1.00 (Reference)	1.00 (Reference)
Moderately isolated	24 848	4418/318 876	1.22 (1.17, 1.28)	1.13(1.08, 1.18)	1.12 (1.07, 1.17)
Most isolated	9965	2386/123 640	1.61 (1.53, 1.70)	1.36(1.29, 1.44)	1.33 (1.26, 1.40)
Loneliness					
No	55 908	9406/720 284	1.00 (Reference)	1.00 (Reference)	1.00 (Reference)
Yes	4423	1013/55 603	1.30 (1.21, 1.38)	1.16 (1.09, 1.25)	1.10 (1.03, 1.17)

Model 1, adjusted for age, sex, ethnicity, education, Townsend deprivation index, and assessment centers;

Model 2, included model 1 plus physical activity, smoking status, alcohol consumption, healthy diet score, and body mass index.

Model 3, plus model 2 and history of depression, history of CVD, history of hypertension, history of diabetes, history of dyslipidemia, and eGFR.

CKD: chronic kidney disease; CVD: cardiovascular disease; eGFR: estimated glomerular filtration ratio.

**Figure 4.**
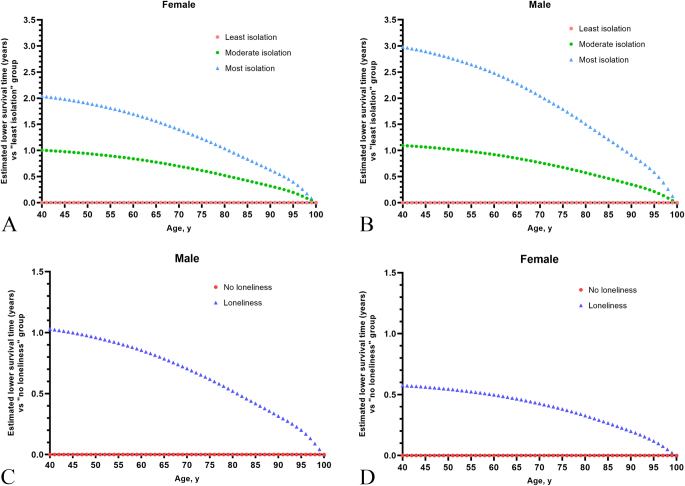
The estimates of cumulative survival time from 40 years of age onward among women and men with different status of loneliness and social isolation. (A) Cumulative survival time estimates for men with different social isolation status. (B) Cumulative survival time estimates for men with different social isolation status. (C) Cumulative survival time estimates for women with different loneliness status. (D) Cumulative survival time estimates for men with different loneliness status.

Life expectancy analyses demonstrated a significant reduction in survival for CKD patients with higher levels of social isolation and loneliness (Fig. 4). At the age of 40 years, the most isolated CKD patients had a reduction in life expectancy of 2.04 years for females and 2.98 years for males, compared to the least isolated group. Moderately isolated patients experienced a reduction of 1.00 years for females and 1.09 years for males. CKD patients reporting loneliness had a life expectancy reduction of 0.58 years for females and 1.03 years for males.

### Secondary analyses

Subgroup analyses and interaction tests revealed no significant effect modification for the majority of subgroups (Supplemental Digital Content Tables 10–13, available at: http://links.lww.com/JS9/F168). Notable interactions were observed between social isolation and the Townsend Deprivation Index, smoking status, and alcohol consumption (Supplemental Digital Content Tables 10 and 12, available at: http://links.lww.com/JS9/F168). Our primary findings remained robust in sensitivity analyses accounting for competing mortality risks using the Fine–Gray method (Supplemental Digital Content Table 14, available at: http://links.lww.com/JS9/F168) and after excluding incident CKD cases or deaths within the first 2 years of follow-up (Supplemental Digital Content Tables 15–18, available at: http://links.lww.com/JS9/F168).

## Discussion

This large-scale prospective cohort study, involving 406 332 individuals without pre-existing CKD and 60 331 patients with CKD, provided compelling evidence that social isolation and loneliness are significant psychosocial risk factors for both the incidence and prognosis of CKD. Individuals with the highest levels of social isolation exhibited a 21% increased risk of developing CKD, while those reporting loneliness had a 26% higher risk. Among CKD patients, the most socially isolated individuals and those experiencing loneliness faced 33% and 10% greater risks of mortality, respectively. Moreover, social isolation, but not loneliness, demonstrated a significant interaction with genetic susceptibility to CKD incidence.

Our findings offer novel insights into the previously underexplored relationship between social isolation, loneliness, and CKD risk, identifying substantial associations in the general population that may inform future prevention and intervention strategies. Previous research has focused primarily on specific subgroups, such as individuals with diabetes^[19,36]^ or those at risk of rapid renal function decline[18]. Our study broadens these findings to a general population context. A prospective study involving 3031 participants aged ≥45 years with baseline eGFR ≥60 ml/min/1.73 m^2^ found that individuals with high levels of social isolation had an 81% higher risk of rapid renal function decline and an 84% higher risk of developing CKD, compared to those with low levels of isolation[18]. Similarly, a cohort study of 6972 type 2 diabetes patients reported that those in the highest tertile of social network score (indicating less isolation) exhibited an 11% lower risk of developing CKD compared to those in the lowest tertile[36]. Additionally, another study on diabetes patients revealed that a higher loneliness scale score, but not social isolation, was significantly associated with a 25% CKD risk[19]. In contrast, our study encompasses a broader population, indicating that both social isolation and loneliness are independently associated with an increased CKD risk, underscoring the importance of addressing these psychosocial factors in public health and clinical strategies aimed at CKD prevention. Although some of the observed hazard ratios were modest – for example, the HR of 1.04 for moderate social isolation and CKD incidence – they were statistically significant and directionally consistent across models. From a clinical perspective, such small effect sizes should be interpreted with caution, as they may be subject to residual confounding or measurement error. However, considering the high prevalence of social disconnection in the general population and the chronic, progressive course of CKD, even modest increases in risk could translate into a substantial public health burden. This highlights the importance of addressing social disconnection not only at the individual level but also through population-based preventive strategies.

Importantly, we did not observe a significant interaction effect between these two factors, and individuals experiencing both social isolation and loneliness had the highest risk of CKD. This highlights the distinct yet complementary roles that social isolation (which reflects the objective reduction in interpersonal interactions) and loneliness (which reflects the subjective feeling of isolation) play in the development of CKD. These findings underscore the importance of addressing both objective and subjective aspects of social connections when considering preventive strategies for CKD. Our subgroup analyses revealed that the adverse impacts of social isolation and loneliness on CKD were more pronounced among individuals experiencing socioeconomic deprivation and those who smoked. This observation aligns with the cumulative disadvantage theory, which posits that intersecting social and behavioral risk factors exacerbate disease susceptibility. These findings underscore the necessity for targeted psychosocial interventions within a comprehensive framework that simultaneously addresses smoking and socioeconomic disparities. Our findings have meaningful implications for both clinical practice and public health. From a clinical standpoint, integrating brief psychosocial screening tools into routine assessments – such as single-item measures of loneliness or social connectedness – could help identify individuals at elevated risk of CKD progression, particularly in primary care or nephrology settings. Such individuals may benefit from more intensive follow-up, early lifestyle interventions, or referrals to support services. At the public health level, population-based strategies aimed at reducing social disconnection may offer scalable opportunities to reduce the CKD burden. Community programs that promote social participation (e.g., peer support groups, group-based exercise, or volunteer activities), as well as digital platforms that facilitate virtual social engagement for homebound or elderly individuals, may help mitigate the health impacts of isolation. Targeting socially isolated populations – especially in socioeconomically deprived communities – could be an important lever for improving long-term kidney health outcomes and narrowing health disparities.

To the best of our knowledge, this study represents the first prospective investigation into the joint association of social isolation, loneliness, and genetic risk score on the incidence of CKD, thereby linking psychosocial determinants and genetic predisposition to the onset of CKD. We observed that social isolation significantly interacted with genetic susceptibility to CKD incidence, suggesting a potential gene-environment interaction. Specifically, individuals with high genetic risk and high levels of social isolation may require tailored interventions to mitigate CKD risk. However, the association between loneliness and CKD remained consistent across genetic risk strata with an insignificant interaction, implying that loneliness is an independent risk factor for CKD, regardless of genetic background. This divergence indicates that the mechanisms underlying social isolation and loneliness may differ. Social isolation, considered an objective social condition, may interact with genetic susceptibility through biological pathways, such as systemic inflammation and dysregulation of the hypothalamic–pituitary–adrenal (HPA) axis^[37,38]^, thereby exacerbating renal risk. Conversely, loneliness, as a subjective experience, may influence outcomes through psychological or behavioral mechanisms that are not necessarily contingent on genetic predisposition^[39,40]^. These findings highlight the importance of differentiating between structural and perceived social disconnection when assessing the risk of CKD and formulating prevention strategies.

Few studies have explored the relationship between psychosocial factors and CKD prognosis. Our findings provide the first evidence linking social isolation and loneliness to increased all-cause mortality risk and reduced life expectancy among CKD patients, aligning with previous meta-analyses that have established similar associations in the general population^[12,17,41]^. Recent pooled analyses of 90 prospective cohort studies encompassing over 2 million individuals found that social isolation and loneliness were associated with 32% and 14% higher risks of all-cause mortality, respectively[15]. In support of our results, data from the Dialysis Outcomes and Practice Patterns Study (1996–2008), which included 32 332 hemodialysis patients from 12 countries, revealed that elevated mortality rates were observed among patients reporting health-related social restrictions, social isolation, perceived burden, or dissatisfaction with family support[42]. Our study further contributes to this body of literature by demonstrating that CKD patients with the highest levels of social isolation experienced a 33% increased mortality risk, while those reporting loneliness had a 10% higher risk. These findings emphasize the critical need to address psychosocial factors in CKD management to enhance both survival and quality of life for affected individuals. Our study elucidates that social isolation and loneliness independently contribute as risk factors to the incidence and mortality of CKD, underscoring the critical necessity for interventions at both clinical and public health levels. From a surgical standpoint, the results of our study have significant implications, especially for patients with advanced CKD who are candidates for dialysis access procedures or kidney transplantation. Social isolation and loneliness may adversely affect treatment adherence, elevate postoperative complications through stress-related physiological mechanisms, and hinder recovery due to insufficient social support. In the realm of kidney transplantation, preoperative psychosocial evaluation is increasingly acknowledged as a crucial element of surgical candidacy, given that inadequate social support has been linked to poorer graft outcomes and elevated rejection rates[43]. Patients undergoing hemodialysis or peritoneal dialysis derive substantial benefits from robust social networks, which facilitate adherence to intricate treatment regimens and bolster psychological well-being[42]. Consequently, the incorporation of psychosocial screening and interventions into perioperative care pathways has the potential to enhance not only patient survival but also quality of life and postoperative outcomes. These considerations highlight the significance of our findings in guiding multidisciplinary approaches in surgical nephrology and emphasize the necessity of psychosocial support as an integral component of comprehensive CKD management.

Effective strategies may encompass routine psychosocial risk assessments during nephrology consultations to identify patients at elevated risk; the incorporation of social workers or psychological counselors into multidisciplinary care teams to furnish emotional and social support; and the establishment of group-based peer support or structured social engagement programs aimed at enhancing mental well-being. These interventions should be tailored to align with the cultural context and the available healthcare resources. In settings with limited resources, the utilization of community health workers or digital health platforms may facilitate the expansion of such interventions. Further research is imperative to assess the feasibility, cost-effectiveness, and long-term outcomes of these strategies within real-world clinical practice.

Life expectancy analyses further highlight the profound impact of social isolation and loneliness on survival among CKD patients, which have never been reported before. At age 40, the most isolated individuals had a reduced life expectancy of 2.04 years for females and 2.98 years for males compared to the least isolated group. Similarly, loneliness was associated with life expectancy reductions of 0.58 years for females and 1.03 years for males. These findings provide novel insights into the tangible consequences of social isolation and loneliness on life expectancy, highlighting the critical public health implications of these psychosocial factors. Sensitivity analyses reinforced the robustness of our findings across various subgroups, showing that associations between social isolation, loneliness, and both CKD incidence and mortality remained consistent.

The mechanisms underlying the associations between social isolation, loneliness, and CKD development and progression are multifaceted. CKD constitutes not merely a medical condition but also a substantial social challenge, influencing patients’ lives in a variety of complex dimensions. The chronic nature of CKD and its associated treatments can significantly disrupt patients’ social roles, as they frequently encounter limitations in their daily activities and social interactions. This disruption is further intensified by the burdens inherent in managing the disease, which include frequent medical appointments, dietary restrictions, and the physical and emotional challenges posed by treatment modalities such as dialysis^[19,44,45]^. Research indicates that a significant number of individuals with CKD experience loneliness and social isolation, factors that may exacerbate their mental health challenges and overall well-being[22]. Psychological stress associated with loneliness and social isolation may also exacerbate existing health conditions, promoting CKD progression[46]. Moreover, physiological pathways, such as increased inflammation, may play a critical role. Socially isolated individuals exhibit elevated levels of pro-inflammatory cytokines and markers of systemic inflammation, such as soluble urokinase plasminogen activator receptors, which are implicated in CKD pathogenesis.^[47,48]^ Additionally, disruptions in the gut–immune–brain axis and neurochemical systems have been linked to the adverse health effects of social isolation, suggesting potential targets for future research[49]. In rodent models, extended periods of isolation result in increased levels of circulating pro-inflammatory cytokines, including interleukin-6 (IL-6) and tumor necrosis factor-α (TNF-α), and lead to the upregulation of inflammatory gene expression in leukocytes[37]. These animals demonstrate dysregulation of the gut–immune–brain axis, characterized by decreased microbial diversity and heightened intestinal permeability, which exacerbates systemic inflammation[49]. Clinically, higher baseline levels of IL-6 and C-reactive protein predict faster eGFR decline and progression to end-stage renal disease in human CKD cohorts, and elevated circulating TNF receptors 1 and 2 have been shown to forecast ESRD in diabetic patients[50]. Collectively, these findings substantiate a pathway through which social isolation exacerbates the risk of CKD via mechanisms involving inflammation and neuroimmunological alterations. Despite these advances, further studies are needed to clarify the biological mechanisms through which social isolation and loneliness influence kidney health.

Despite the strengths of our study, including its large sample size, robust statistical analyses, and adjustment for numerous confounders, several limitations warrant consideration. First, the dynamic nature of social isolation and loneliness was not captured during follow-up, potentially leading to misclassification bias that may underestimate the true associations. Second, the UK Biobank’s low response rate (5.47%) during the baseline enrollment may introduce potential selection bias. However, recent evidence suggests that risk factor associations in the UK Biobank are broadly generalizable, mitigating concerns about selection bias[51]. This selection bias likely led to an underestimation of the true effect sizes associating social isolation and loneliness with the risk of CKD. Consequently, our findings may represent conservative estimates of the actual associations, particularly in more vulnerable populations. Third, both social isolation and loneliness were measured only at baseline, without accounting for temporal changes. As social connections may fluctuate over time and interact with disease progression, future research should incorporate repeated assessments to capture dynamic exposure patterns and better evaluate causal pathways. Fourthly, the predominantly European Caucasian composition of the cohort may limit the generalizability of our findings to other populations with different genetic and cultural backgrounds. Fourth, although we have considered many confounding factors, residual confounding cannot be completely avoided. Finally, as an observational study, causality cannot be definitively established. To overcome the limitations inherent in our current design, future research should incorporate longitudinal methodologies with repeated assessments of social isolation and loneliness over time. This approach would facilitate more precise modeling of dynamic exposure patterns and allow for a more comprehensive understanding of the cumulative psychosocial impact on renal health. Furthermore, broadening recruitment efforts to encompass multiple centers across diverse socioeconomic and geographic contexts would enhance the external validity of the findings, thereby ensuring broader applicability to diverse populations, especially those at an elevated risk for both social disconnection and CKD.

## Conclusion

Our study provides novel and compelling evidence that social isolation and loneliness are significant, independent risk factors for CKD incidence and mortality. These findings underscore the critical importance of addressing psychosocial determinants in CKD prevention and management. Public health initiatives and clinical interventions aimed at mitigating social isolation and loneliness hold the potential to reduce the burden of CKD and improve survival outcomes for affected individuals. Future research should focus on elucidating the biological mechanisms underlying these associations and developing targeted strategies to mitigate their impact on kidney health.

## Data Availability

Data from the UK Biobank are available to all researchers upon making an application. We thank all UK Biobank participants and staff.

## Research registration unique identifying number (UIN)

The UK Biobank study received approval from the North West Multi-centre Research Ethics Committee (reference number 21/NW/0157), and all participants provided written informed consent to participate.
